# Differential gene expression analysis of common target genes for the detection of SARS-CoV-2 using real time-PCR

**DOI:** 10.1186/s13568-022-01454-2

**Published:** 2022-09-02

**Authors:** Reza Valadan, Soheila Golchin, Reza Alizadeh-Navaei, Mohammadreza Haghshenas, Mehryar Zargari, Tahoora Mousavi, Mohammad Ghamati

**Affiliations:** 1 Molecular and Cell Biology Research Center, Faculty of Medicine, Mazandaran University of Medical Sciences, Sari, Iran; 2 Department of Immunology, Faculty of Medicine, Mazandaran University of Medical Sciences, Sari, Iran; 3 Gastrointestinal Cancer Research Center, Non-communicable Diseases Institute, Mazandaran University of Medical Sciences, Sari, Iran; 4 Department of Microbiology, Molecular and Cell-Biology Research Center, Faculty of Medicine, Mazandaran University of Medical Sciences, Sari, Iran; 5 Department of Clinical Biochemistry and Genetics, Molecular and cell biology research center, Faculty of Medicine, Mazandaran University of medical sciences, Sari, Iran; 6 Molecular and Cell Biology Research Center, Hemoglobinopathy Research Institute, Mazandaran University of Medical Sciences, Sari, Iran; 7 Medical student, Mazandaran University of Medical Sciences, Sari, Iran

**Keywords:** SARS-CoV-2, RT -PCR assay, RDRP, N, And E genes expressions

## Abstract

COVID-19 currently is the main cause of the severe acute respiratory disease and fatal outcomes in human beings worldwide. Several genes are used as targets for the detection of SARS-CoV-2, including the RDRP, N, and E genes. The present study aimed to determine the RDRP, N, and E genes expressions of SARS-CoV- 2 in clinical samples. For this purpose, 100 SARS-CoV-2 positive samples were collected from diagnostic laboratories of Mazandaran province, Iran. After RNA extraction, the real-time reverse transcription PCR (real-time RT-PCR) assay was performed for differential gene expressions’ analysis of N, E, and RDRP. The threshold cycle (Ct) values for N, RDRP, and E targets of 100 clinical samples for identifying SARS-CoV-2 were then evaluated using quantitative real-time PCR (qRT-PCR). This result suggests N gene as a potential target for the detection of the SARS-CoV‐2, since it was observed to be highly expressed in the nasopharyngeal or oropharynges of COVID-19 patients (P < 0.0001). Herein, we showed that SARS-CoV- 2 genes were differentially expressed in the host cells. Therefore, to reduce obtaining false negative results and to increase the sensitivity of the available diagnostic tests, the target genes should be carefully selected based on the most expressed genes in the cells.

## Introduction

COVID-19, which is currently known as the global pandemic of Coronavirus, is responsible for the severe acute respiratory disease and fatal outcomes in human beings worldwide (Korber et al. 2020). Coronaviruses as a group of enveloped viruses with positive-sense single-stranded RNA belong to the family Coronaviridae, which are able to spread between humans and animals (Holshue et al. 2020).

Unlike HCoV-229E, HCoV-OC43, HCoV-NL63, HCoV-HKU1 to cause moderate upper respiratory infection in human (Hu et al. 2014), three previous epidemics of β-coronaviruses such as Middle East respiratory syndrome-related Coronavirus (MERS-CoV), severe acute respiratory syndrome Coronavirus (SARS-CoV), and severe acute respiratory syndrome Coronavirus 2 (SARS-CoV-2), have been potentially associated with acute respiratory distress syndrome (ARDS) with about 35.5%, 9.6%, and 6.76% mortality rates, respectively (Lu et al. 2020).

SARS-CoV-2 contains open reading frames (ORFs) that encode four structural proteins, including S-spike, M-membrane, E-envelope, and N-nucleocapsid. Of note, several genes are used as the targets gene for detection of SARS-CoV-2 such as the (RDRP and S), (N and S), and E genes. In this regard, studies have previously shown that N protein is produced in large quantities in infected cells, which is related to the processes of replication, translation, and transcription. Moreover, it causes cell cycle deregulation, consequently inhibiting interferon production and inducing apoptosis (Astuti 2020). The RNA-dependent RNA polymerase, called ORF1ab, is responsible for viral transcription and replication. Therefore, RT-PCR based 2 target genes (ORF1ab and N) are the crucial targets for SARS-CoV-2 detection (Shen et al. 2021).

In order to have the best RT-PCR performance, the components of these targets should be optimized (Tombuloglu et al. 2021). Accordingly, reverse transcription polymerase chain reaction (RT-PCR) using fluorescent dyes is considered as a gold standard method for detecting bacterial and viral nucleic acid (DNA / RNA). RT-q PCR can also be used as a rapid and accurate assay for screening SARS-CoV-2 in throat samples, nasopharyngeal swabs, and feces (Chaimayo et al. 2020). A cohort study has shown that RT-PCR with sensitivity and specificity values of 70% and 95% could detect viruses in patients, even in those showing no symptoms (Arevalo-Rodriguez et al. 2020; Rutuja Sunil and Vasudeo Pandharinath 2021). However, a successful detection of this virus depends on some factors such as test time, early or late detection time, viral load, and sample collection procedure (Vickers 2017).

The ORF1ab/RdRp, E, N, and S genes most commonly used targets for detection of SARS-CoV-2 so, there are some commercial RT-PCR kits for the diagnosis of COVID-19 such as Primer Design (England, RdRp), Seegene (Korea, RdRp, N, E), CerTest Biotec (Spain, ORF1ab, N), Altona Diagnostics (Germany, S, E), BGI (China, ORF1ab), KH Medical (Korea, RdRp, S), and R-Biopharm AG (Germany, E) with different qualities, which are available to be used for the diagnosis of SARS-CoV-2 (Puck et al. 2020). According to this point that diagnosis of SARS-CoV-2 infection with two or three targets lead to an increase in sensitivity and specificity and avoid a false negative result, so the present study attempted to analyze the RDRP, N, and E genes expressions of SARS-COV- 2 using qRT-PCR through specific primer pairs in the obtained clinical samples.

## Materials and methods

### Simplex primer and probe design

**Fig. 1:**
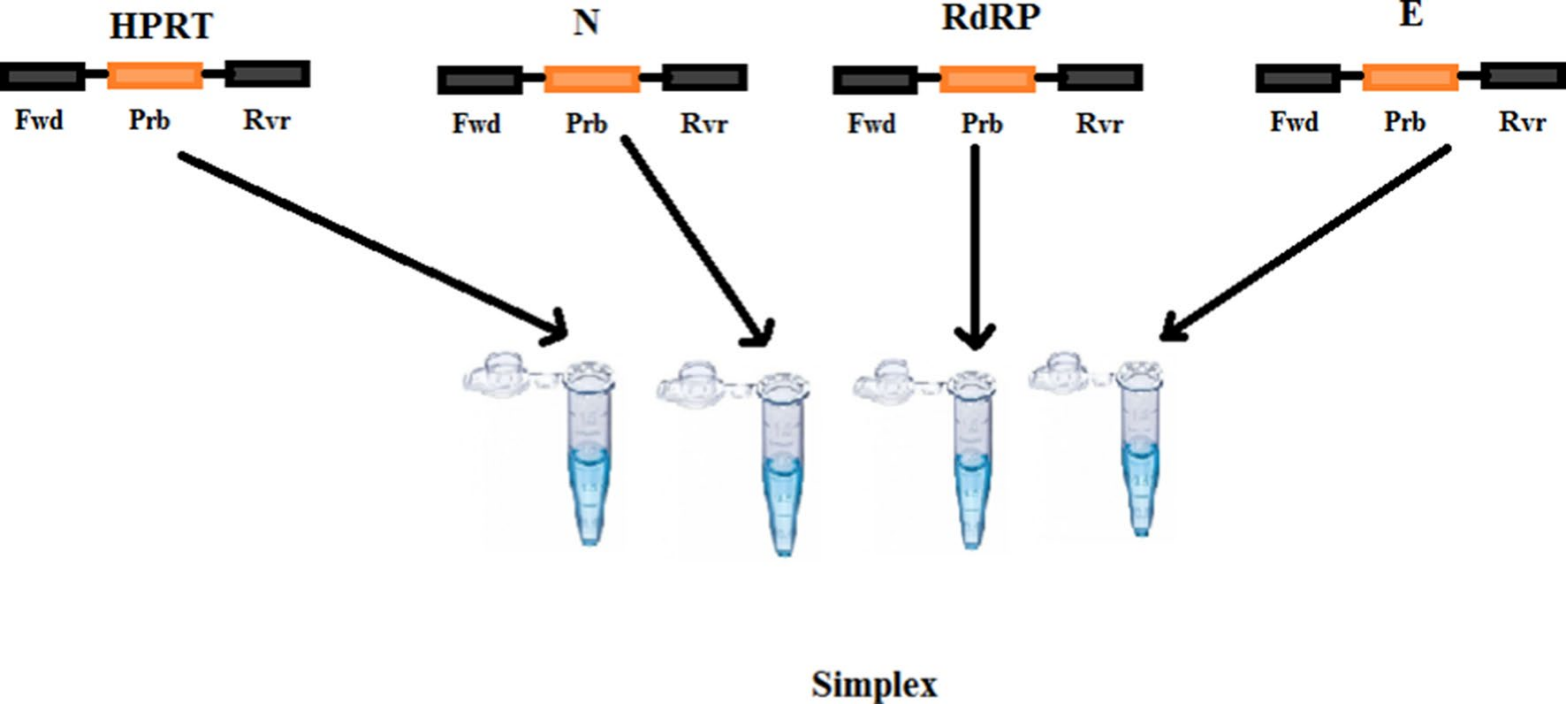
Experimental design of qRT-PCR

The specific qRT-PCR primers and probe for the diagnosis of the target regions of the SARS-​CoV-2 were designed using the following programs: PrimerPooler, PrimerPlex, and Primer3 (Tombuloglu et al. 2021). Moreover, 5’ Fluorescein amidites (FAM)-labeled probe was designed for the SARS-CoV-2 RdRp/ N/RP, as well as Hypoxanthine Phosphoribosyltransferase (HPRT) and Yakkima yellow-labeled probe for the viral E gene, which were then synthesized (Fig. 1). The sequence of each primer or probe is shown in Table 1.

**Table 1:** The sequences and concentrations of primer and probe sets used in the PCR reactions

Target	Sequence(5′-3′)	Label	Reference
2019-nCoV_N1-F	GAC CCC AAA ATC AGC GAA AT	None	(Jung et al. 2020)
2019-nCoV_N1-R	CT GGT TAC TGC CAG TTG AAT CTG	None	(Jung et al. 2020)
2019-nCoV_N1-P	FAM-ACC CCG CAT TAC GTT TGG TGG ACC-BHQ1	FAM, BHQ1	(Bruce et al. 2020)
E_Sarbeco_F1	ACAGGTACGTTAATAGCGT	None	(Corman et al. 2020a)
E_Sarbeco_R1	ATATTGCAGCAGTACGCACACA	None	(Corman et al. 2020a)
E_Sarbeco_P	Yakkima yellow-ACACTAGCCATCCTTACTGCGCTTCG-BHQ1	Yakkima yellow, BHQ1	(Corman et al. 2020a)
2019-nCoV_ RDRP-F1	GTGARATGGTCATGTGTGGCGG	None	(Corman et al. 2020a)
2019-nCoV_ RDRP-R	CARATGTTAAASACACTATTAGCATA	None	(Corman et al. 2020a)
2019-nCoV_ RDRP-P	FAM-CAGGTGGAACCTCATCAGGAGATGC-BHQ1	FAM, BHQ1	(Corman et al. 2020a)
HPRT-F	GGACTAATTATGGACAGGACTG	None	(Valadan et al. 2015a)
HPRT-R	GCTCTTCAGTCTGATAAAATCTAC	None	(Valadan et al.2015a)
HPRT-P	FAM-CCTCCCATCTCCTTCATCACATCTC–BHQ1	FAM, BHQ1	(Valadan et al. 2015a)
RP-F	AGA TTT GGA CCT GCG AGC G	None	(Gregianini et al. 2019)
RP-R	GAG CGG CTG TCT CCA CAA GT	None	(Gregianini et al. 2019)
RP-P	FAM – TTC TGA CCT GAA GGC TCT GCG CG – BHQ-1	FAM, BHQ1	(Gregianini et al. 2019)

### RNA extraction from the clinical samples

The study was approved by the Mazandaran University of Medical Sciences, Iran, with the number IR.MAZUMS.REC.1399.8671. For the purpose of this study, Nasopharyngeal and oropharyngeal swabs were collected from symptomatic patients, immediately diluted with viral transfer medium (VTM), and finally transferred to the COVID-19 laboratory at Mazandaran University of Medical Sciences for the detection of SARS-CoV-2. RNA extraction was performed in 100 positive samples using the RNJia virus kit (Jivan, Iran) in terms of the manufacturer’s instructions. Subsequently, the differential gene expressions of N, E, and RdRp were performed using qRT-PCR.

### Real-time RT-PCR assay

In this study, 20-µL reaction containing 4 µL of RNA, 10 µL of one step RT-PCR kit(add bio, korea), 2 µL of enzyme mixture, 0.5 µL of forward and reverse primers, 0.5 µL of each probe, and RNase/DNase-free ddH2O up to 20 µL, was setup. Final primers and probes concentrations in the reaction were adjusted using the following steps:

1.0.25 pM for RdRP-F, and 0.25 pM for RdRP-R.
2. 0.25 pM for E-F, and 0.25 pM for E-R.
3. 0.25 pM for N-F, and 0.25 pM for N-R.
4. 0.25 pM for HPRT-F, and 0.25 pM for HPRT-R.

The reaction was dispensed in 96-well microplates (MicroAmp™ Fast Optical 96-well reaction Plate 0.1 mL, Applied Biosystems) and then sealed with optical film (MicroAmp™ Optical Adhesive Film, Applied Biosystems). Of note, a negative control reaction (RNase/DNasefree ddH2O) was used to check the presence of any contamination. In addition, HPRT and RP genes were used as internal controls (Valadan et al. 2015b).

Thereafter, Quantitation experiments were conducted using RT-PCR instrument (StepOne™ Real-Time PCR System).

As well, qPCR was performed as follows:

1. Reverse transcription was performed for 20 min at 50 °C,
2. Inactivation of the reverse transcriptase was done for 10 min at 95 °C.
3. PCR amplification was performed with 40 cycles for 15 s at 95 °C and for 30 s at 58 °C using StepOne^™^ Real-Time PCR.

### Statistical analysis

The obtained results were examined by determining the amplification curve of the target gene and the housekeeping gene. Continuous variables are indicated as means (standard deviation, SD). All the statistical analyses were performed using GraphPad Prism 8 software and p-values less than 0.001 were considered as statistically significant.

## Results

In the present study, 100 respiratory samples were collected from nasopharyngeal (NP) and throat swabs in health-care centers of Mazandarn, Iran, from December 2020 to September 2021. Thereafter, Real-time RT-PCR, using E, RDRP, and N targets, was performed for genome detection of SARS-CoV-2. Firstly, all the primers and probes were analyzed by simplex qRT-PCR. Prior to preparing the reactions, the qRT-PCR instrument was properly calibrated in order to achieve the best fluorescent signal. The simplex reactions were then performed in triplicate for three viral E, N, and RDRP genes as well as internal control genes (HPRT and RP). The criteria for the diagnosis of positive, negative, and suspicious COVID-19 samples were as follows: (0 < Ct < 37.00), (NO Ct or Ct ≥ 40.00), and (37.00 ≤ Ct < 40.00), respectively.

**Fig. 2:**
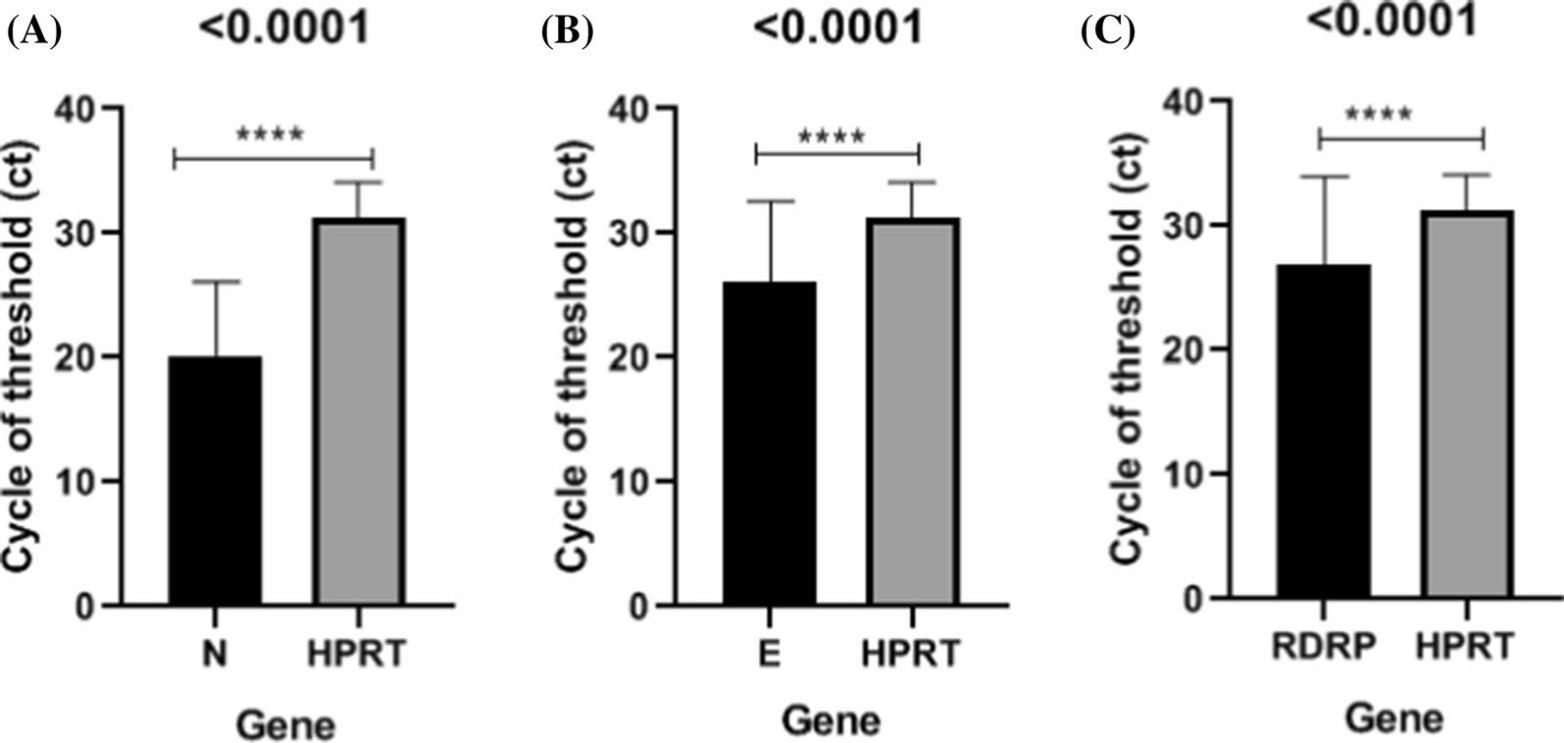
Cycle threshold (Ct) value of qRT-PCR. HPRT gene was used as an internal control.** A** Comparison of N target and HPRT,** B** Comparison of E target and HPRT, and** C** Comparison of RDRP target and HPRT. A significant difference is indicated by *P < 0.05. **** = (< 0.0001).

**Fig. 3:**
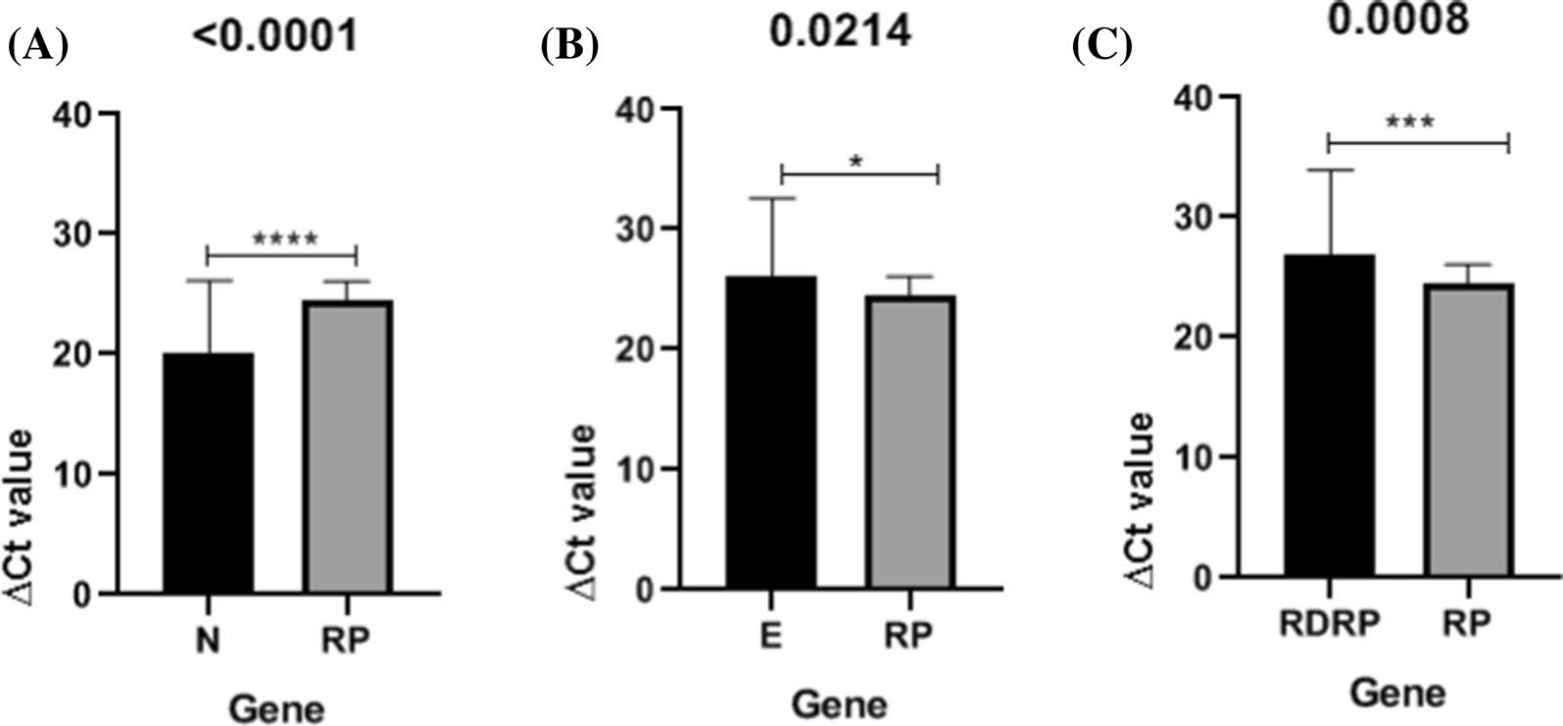
Cycle threshold (Ct) value of qRT-PCR. RP gene was used as an internal control.** A** Comparison of N target and RP,** B** Comparison of E target and RP, and** C** Comparison of RDRP target and RP. A significant difference is indicated by *P < 0.05. **** = (< 0.0001).

**Fig. 4:**
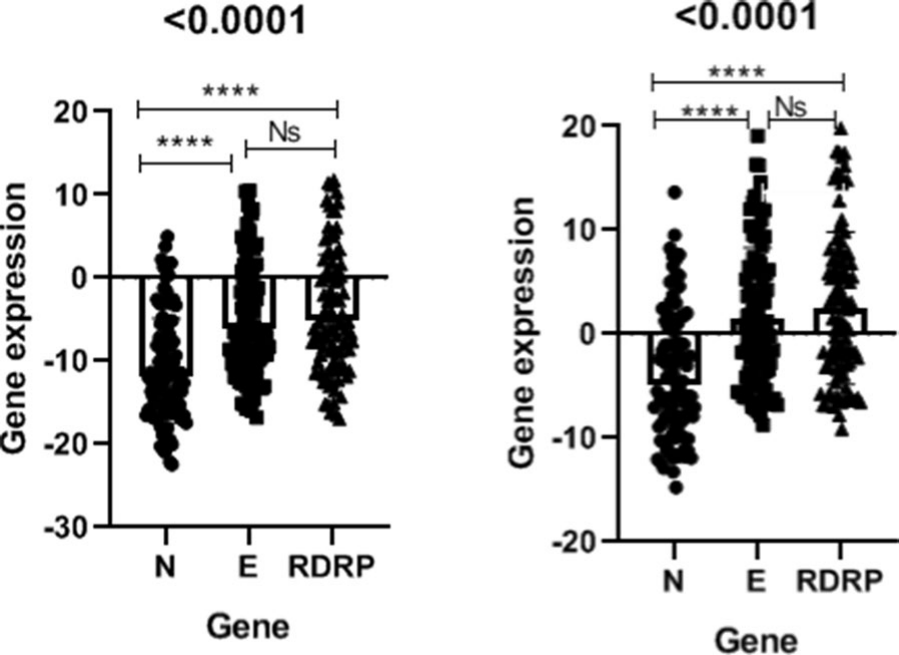
Comparison of the cycle threshold (∆Ct) value of SARS-COV-2 expression.** A** HPRT gene was used as an internal control. A significant difference is indicated by *P < 0.05. ****, Ns = Not significant (0.611).** B** RP gene was used as an internal control. A significant difference is indicated by *P < 0.05. ****, Ns = Not significant, (0.608).

The average cycle threshold (Ct) and ∆Ct value with standard deviations (SD) are shown in Tables 2 and 3, and the comparative Ct performances of each assay are shown in Figs. 2 and 3. In this research, HPRT and RP genes were used as internal controls. Indeed HPRT and RP had significantly increased expression level compared to other targets (including N, E, and RDRP) (P < 0.0001). Our findings showed that no detectable difference exists between HPRT and RP internal controls. According to the comparison of ∆Ct values among N, E, and RDRP targets, the N gene expression level was found to be higher than that of E and RDRP genes. (P < 0.0001). As shown in Fig. 4, there is no significant difference between E and N targets (0.611). The result of our study suggest N gene as the most sensitive target compared to E and RDRP for SARS-CoV-2 detection using RT-PCR.

**Table 2:** The Ct value emerged in RT-PCR assay for the SARS-COV-2

Characteristics	Result
Ct-value of E Mean ± SD (min, max)	26 ± 6.53 (14.24, 39.24)
Ct-value of N Mean ± SD (min, max)	20.19 ± 5.93 (9.72, 33.82)
Ct-value of RDRP Mean ± SD (min, max)	26.92 ± 7.04 (13.85, 40)
Ct-value of HPRT Mean ± SD (min, max)	31.34 ± 2.75 (26.94, 40)
Ct-value of RP Mean ± SD (min, max)	24.43 ± 1.63 (20.56, 27.94)

**Table 3:** The ∆Ct value emerged in RT-PCR assay for the SARS-COV-2.

Characteristics	∆Ct value (HPRT control)	∆Ct value (RP control)
E gene Mean ± SD (min, max)	−5.34 ± 6.76 (−15.15, 0)	1.57 ± 6.74 (−8.71, 19.07)
N gene Mean ± SD (min, max)	−11.15 ± 6.28 (−22.45, 0.43)	−4.23 ± 6.13 (−13.23, 13.65)
RDRP gene Mean ± SD (min, max)	−4.42 ± 7.27 (−16.91, 11.42)	2.49 ± 7.32 (−6.84, 17.59)

## Discussion

In this study, Ct values for the N, RDRP, and E targets were evaluated using qRT-PCR in order to detect SARS-CoV-2 in 100 clinical samples. It was observed that N gene has less Ct values (23.73 ± 6.99) than those of E and RDRP. Moreover, our results show a significant difference among the E, N, and RDRP groups.

The diagnosis of SARS-CoV-2 using molecular tests is known as the gold standard method for the diagnosis of COVID-19 infection. Of note, the RT-PCR is a sensitive assay for the detection of SARS-CoV-2 RNA in clinical specimens (Chaimayo et al. 2020). The study showed that after the onset of the disease’s symptoms, the SARS-CoV-2 viral load can be immediately observed in the upper respiratory tract and the antigen can also be detected in the first phase. However, some factors such as clinical manifestations, duration of disease to laboratory test, type of clinical sample, and sample collection procedure (technique process) can be effective on interpreting the results (Zou et al. 2020).

In general, many developed laboratory methods use various tools, reagents, and targets in order to identify SRRS-COV-2 (LeBlanc et al. 2020). RDRP, E, and N are three targets proposed by WHO for the SARS-COV-2 identification (Corman et al. 2020a). As well, the E gene is the first line screening, the RDRP gene is used as confirmatory test, and the N gene is used for a confirmatory testing, all of which are used in identifying the coronavirus. A previous study has shown that the RdRP_SARSr-P2 target could be specific for the coronavirus, and other probes are suitable for the detection of other types of coronavirus, and if false positive results are obtained regarding the diagnosis of Covid-19, it may possibly indicate that patients with mild symptoms are infected with other types of corona virus (Kakhki et al. 2020b). Besides, evidence suggests that other targets such as ORF8 and specific primers / probes, may act as additional confirmatory tests in the diagnosis of SARS-COV-2 (kamali Kakhki et al. 2020a).

Houda et al. in their study have evaluated three genes of RDRP, N, and E in 187 COVID-19 samples and found gene expression as 22% and 40% in N and N, E genes, respectively. They have also shown that 6% of patients with both E and N genes and 14% of those with N gene still remained positive after a 12-day treatment period (Benrahma et al. 2020). In addition, a study of 114 respiratory specimens has revealed that the N Ct value was more specific for laboratory diagnosis of SARS-CoV-2 (Abbasi et al. 2022).

However RT-qPCR has a high levels of specificity and sensitivity, but sensitivity of COVID-19 RT-PCR diagnostic kits could be associated to the specimen conditions such as transportation or storage, sample preservation times, and the quality of the kits (Bezier et al. 2020). COVID-19 RT-PCR diagnostic kits with high analytical specificity and sensitivity could help reduce the impact of false-negative results and significantly improve the identification of COVID-19 patients (Shen et al. 2021).

Another study has shown that the one-​step real-time RT-PCR can detect SARS-CoV-2 RNA in clinical specimens with a low detection sensitivity (Michel et al. 2021). Since January 2020, protocols, tests, and reagents have been developed and introduced for the detection of SARS-COV-2. These laboratory tests that use SARS-CoV-2 RNA for the detection of COVID-19, were compared with commercial kits. A previous study using RT-PCR and two primers (N1 and N2) for SARS-COV-2 identification (Shirato et al. 2020) has shown that N2 primer has high specificity and sensitivity in this regard. These primers were also assessed using the following commercial kits: LN S & W-E, LN S & W-N, and LMW & RDRP (Hoehl et al. 2020). The results showed that the commercial LN S & W-N kit containing N primer was able to detect the virus better than the LN S & W-E (25 copies detected) and LMW & RDRP kits. It was observed that the LN S & WE targets are strongly conserved in the E gene region on SARS-COV and SARS-COV-2, while the N2 targets are a single region of N gene on SARS-COV-2 virus, so N2 is highly sensitive and specific for the detection of SARS-CoV-2 (Corman et al. 2020b).

This study showed that selection of different targets with high expression lead to increased sensitivity of diagnostic kits, therefore, to reduce false negative results and to increase the sensitivity, diagnostic tests should be designed based on the targets that have the most differential expression. Correspondingly, RT-PCR method using of N, E, and RDRP targets is known as a reliable and accurate method for SARS-CoV-2 identification that can be used in infection’s prevention and control, and in diagnostic laboratories and medical centers.

## Data Availability

The data and materials are mentioned in the manuscript.
